# Role of Forkhead Box O Proteins in Hepatocellular Carcinoma Biology and Progression (Review)

**DOI:** 10.3389/fonc.2021.667730

**Published:** 2021-05-27

**Authors:** Shaojie Yang, Liwei Pang, Wanlin Dai, Shuodong Wu, Tengqi Ren, Yunlong Duan, Yuting Zheng, Shiyuan Bi, Xiaolin Zhang, Jing Kong

**Affiliations:** ^1^ Department of General Surgery, Shengjing Hospital of China Medical University, Shenyang, China; ^2^ Innovation Institute of China Medical University, Shenyang, China

**Keywords:** forkhead box O 1, forkhead box O 3, forkhead box O 4, forkhead box O 6, hepatocellular carcinoma

## Abstract

Hepatocellular carcinoma (HCC), the most common type of malignant tumor of the digestive system, is associated with high morbidity and mortality. The main treatment for HCC is surgical resection. Advanced disease, recurrence, and metastasis are the main factors affecting prognosis. Chemotherapy and radiotherapy are not sufficiently efficacious for the treatment of primary and metastatic HCC; therefore, optimizing targeted therapy is essential for improving outcomes. Forkhead box O (FOXO) proteins are widely expressed in cells and function to integrate a variety of growth factors, oxidative stress signals, and other stimulatory signals, thereby inducing the specific expression of downstream signal factors and regulation of the cell cycle, senescence, apoptosis, oxidative stress, HCC development, and chemotherapy sensitivity. Accordingly, FOXO proteins are considered multifunctional targets of cancer treatment. The current review discusses the roles of FOXO proteins, particularly FOXO1, FOXO3, FOXO4, and FOXO6, in HCC and establishes a theoretical basis for the potential targeted therapy of HCC.

## Introduction

Primary liver cancer is a common clinical malignant tumor of the digestive system. Hepatocellular carcinoma (HCC), the fourth most common tumor and second most common cause of cancer-associated deaths worldwide, accounts for 90% of primary liver cancer cases (1). Hepatitis virus infection, particularly hepatitis B virus (HBV), is a major risk factor in the progression of the hepatitis B/cirrhosis/HCC axis (2). Sex, age, family history, alanine aminotransferase/aspartate aminotransferase levels, history of smoking and drinking, metabolic syndrome, and combined infection with both hepatitis C virus and human immunodeficiency virus may promote the occurrence of HCC independently or together with HBV (3, 4). Due to the insidious onset, high invasiveness, rapid progress, and difficulty of early diagnosis, most patients with HCC harbor advanced-stage tumors or distant metastases at the time of diagnosis, making these patients unsuitable for local treatment, such as surgery, local ablation, or transcatheter arterial chemoembolization (TACE). Systemic chemotherapy can be used to treat patients with advanced liver cancer without contraindications (1); however, there is no evidence that systemic chemotherapy provides patients with a survival benefit. Targeted therapy has become a major focus of contemporary cancer research. Sorafenib (a kinase inhibitor), the most common targeted drug for the treatment of advanced HCC, has several disadvantages, including serious adverse drug reactions, low survival benefit, low efficacy, high cost, and high drug resistance, which greatly limit the application of this drug in patients with HCC (5). Therefore, new prognostic biomarkers and therapeutic targets are urgently required to improve the survival outcomes of patients with HCC.

The forkhead box (FOX) gene family was originally discovered in *Drosophila* as a genetic mutant (6). Murine activators of hepatic-specific gene expression, hepatocyte nuclear factor (HNF) 3α, HNF3β, and HNF3γ, were homologous to FOX proteins in *Drosophila*, leading to the emergence of the FOX protein family (7). The forkhead domain is an important feature of FOX family proteins, forming a ‘wing-like helix’ structure *via* three α-helices, three β-sheets, and a two-sided loop, with a highly conserved DNA sequence containing 110 nucleotides (6). Moreover, the 19 FOX family proteins identified to date are widely expressed in eukaryotes, ranging from yeast to mammals (6).

**Figure 1:**
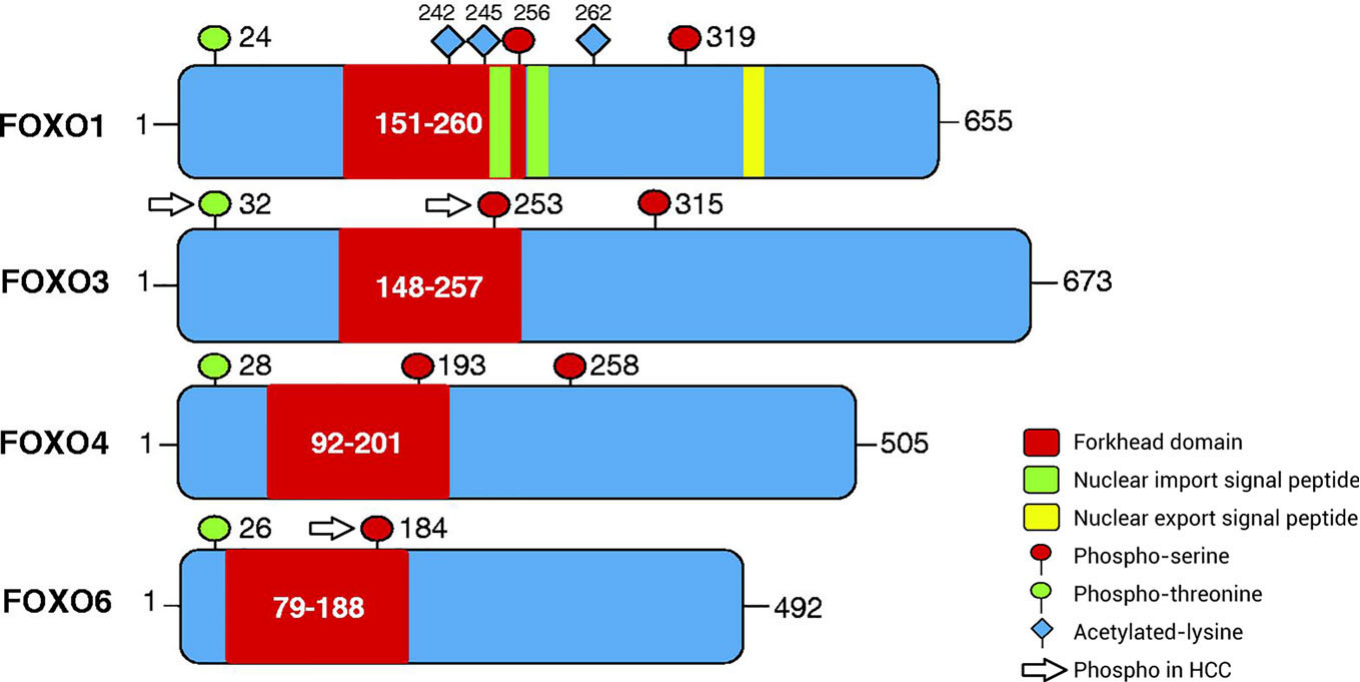
Patterns of post-translational modification of FOXO proteins *via* phosphorylation. The amino acid sequence of FOXO proteins has three phosphorylation sites, including phospho-serine and phospho-threonine sites. The first phosphorylation motif is located after the start codon, the second motif is located in the forkhead domain, and the third motif is located immediately following the forkhead domain. However, the third conserved region of FOXO6 is missing. In addition, FOXO1 has special nuclear import/export signal peptides and acetylated lysine residues. The phosphorylated sites of FOXO proteins in HCC are separate and include ubiquitination and phosphorylation at Ser253 in FOXO3, phosphorylation at Thr32 in FOXO3, and phosphorylation at Ser184 in FOXO6. FOXO, Forkhead box O protein.

The subfamily of FOXO transcription factors, including FOXO1 (also known as forkhead in rhabdomyosarcoma), FOXO3 (also known as forkhead in rhabdomyosarcoma-like 1), FOXO4, and FOXO6, is widely expressed in cells and integrates a variety of growth factors, oxidative stress signals, and other stimulatory signals, thereby inducing the specific expression of downstream signaling molecules and regulating the cell cycle, senescence, apoptosis, oxidative stress, stem cell differentiation, and tumor occurrence and development (8, 9). FOXO proteins can directly affect target genes to alter biological function and can interact with other transcription factors to regulate target genes (7). Under different environmental stimuli, the phosphorylation, acetylation, and ubiquitination of FOXO proteins are important for inducing cell-specific changes (10, 11), among which phosphorylation is the most common mechanism. The amino acid sequence of FOXO proteins has three phosphorylation sites, which are located after the initiation codon, in the forkhead region, and immediately after the forkhead region (**Figure 1**). FOXO proteins can be regulated by the upstream PI3K/PKB phosphorylation pathway and act as a key target of the insulin/insulin-like growth factor (IGF)-1 signaling pathway to regulate cellular functions. The various FOXO phosphorylation pathways also alter the intracellular localization, molecular half-life, DNA binding capacity, and transcriptional activity of FOXO proteins. PKB mediates the phosphorylation of FOXO proteins, thereby driving the translocation of FOXO proteins from the nucleus to the cytoplasm, blocking transcriptional activity, and inhibiting transcription. In the cytoplasm, FOXO proteins can dissociate from 14-3-3 proteins and either return to the nucleus or degrade after being ubiquitinated, resulting in persistent suppression of downstream gene expression (12–15).

Although FOXO proteins are widely expressed, their expression levels and roles vary according to organ. FOXO1 is highly expressed in substantial organs, such as the liver and pancreas, and in several tissue types, such as fat and muscle (16, 17). Both FOXO3 and FOXO4 are widely distributed in tissues, including those of lymph nodes, liver, kidney, heart, and skeletal muscles (16, 17), whereas FOXO6 is prominently expressed in the brain and nervous system (18). Previous studies have shown that FOXO subtypes exhibit differential expression in primary tumor tissues and cells (19–21). Thus, these proteins may function as tumor suppressors or carcinogenic factors, affecting tumor cell function. For example, patients with primary gastric adenocarcinoma exhibit low expression of FOXO3 (19), which is highly expressed in patients with HCC (20, 21). Moreover, the expression levels of FOXO subtypes vary in HCC. However, not all FOXO proteins have been thoroughly investigated.

The current review discusses the expression and roles of FOXO proteins in the occurrence, development, treatment, resistance, and prognosis of tumors, particularly HCC. The review aims to establish FOXO proteins as biomarkers for clinical diagnosis and targets for treatment of HCC.

## Foxo Proteins In Cancer

The proliferation, invasion, and metastasis of malignant tumor cells are important biological processes in the occurrence and development of cancer and are known to affect the treatment and prognosis of patients. Notably, FOXO proteins serve important regulatory roles in tumorigenesis. Although FOXO proteins have been extensively studied as tumor suppressors, with roles in regulating proliferation, the cell cycle, apoptosis, aging, and oxidative stress, they have also been shown to promote tumor growth, metastasis, chemotherapy resistance, and survival.

### Roles of FOXO Proteins in Cancer Cell Proliferation

FOXO proteins can act as negative regulators of tumor cell proliferation by inducing abnormalities in proliferation. By regulating the activities of downstream cell cycle proteins to block the cell cycle, such as cyclinD1 and D2, FOXO proteins induce the expression of specific cell cycle inhibitors, including P21, P27, P15, and P19, arrest cell growth in the G_1_/S phase (22–25), and alter the activities of growth arrest and DNA-damage-inducible α, cyclin G2, and polo-like kinase 1, thereby arresting cells in the G_2_/M phase (26, 27). Mazumdar et al. (28) reported that FOXO1 expression was downregulated in breast cancer and that estrogen E2 could promote the proliferation of tumor cells by inhibiting FOXO1. Moreover, aplysin causes the dephosphorylation of FOXO3a in breast cancer through the PI3K/AKT/FOXO3a signaling pathway and inhibits tumor growth by blocking cell proliferation and promoting apoptosis of tumor cells (29). Chen et al. (30) reported that microRNA (miRNA/miR)-664 acted as an oncogene in osteosarcoma cells and promoted the proliferation of human osteosarcoma cells by inhibiting FOXO4 expression.

FOXO proteins can also act as tumor carcinogens to stimulate tumor cells. Indeed, some FOXO proteins, such as FOXO6, are highly expressed in some cancer tissues and cells, thereby affecting the biological effects of tumors by altering cell proliferation and apoptosis. Li et al. (31) revealed that FOXO6 knockdown inhibited cell proliferation, migration, invasion, and glycolysis in colorectal cancer cells. Additionally, Lallemand et al. (32) demonstrated that overexpression of FOXO6 activated breast cancer cell proliferation and inhibited the endogenous expression of FOXO6 to induce accumulation of cells in the G_0_/G_1_ phase but did not cause apoptosis. Moreover, FOXO3 promotes cell proliferation and inhibits apoptosis in HCC cells, suggesting an association with poor prognosis (20, 33). Furthermore, the upregulation of miR-421 expression elicits carcinogenic effects in nasopharyngeal carcinoma by inhibiting FOXO4 expression, resulting in the inhibition of cell proliferation and resistance to apoptosis (34).

### Roles of FOXO Proteins in Cancer Cell Apoptosis

In human cells, activation of tumor-suppressor genes can regulate apoptosis, thereby inhibiting tumorigenesis. FOXO proteins serve important roles in apoptosis induced by tumor suppressor genes (**Table 1**). For example, FOXO proteins can upregulate BCL-xL through interaction with BCL-6 and inhibit proliferation (35). Additionally, FOXO proteins regulate exogenous apoptosis pathways by modulating the activities of Fas ligand (FasL), tumor necrosis factor-related apoptosis-inducing ligand (TRAIL), and other apoptotic factors (36). Alternatively, FOXO proteins act on the BCL-2 family (BIM/P53) to regulate endogenous apoptotic pathways (37). Courtois-Cox et al. (38) suggested that abnormal activation of RAS could trigger senescence by inhibiting the negative feedback loop of RAS and PI3K, resulting in the activation of FOXO1 and FOXO3. de Keizer et al. (22) revealed that BRAFV600E induced phosphorylation of FOXO4 at sites Thr223, Ser226, Thr447, and Thr451, leading to p21Cip1 expression and causing oncogene-induced apoptosis. Moreover, Wang et al. (39) demonstrated that FOXO4 expression was decreased in clear cell renal carcinoma cells and that overexpression of FOXO4 significantly increased apoptosis rates and expression levels of BIM, BCL-2, BAX, and cytochrome c in clear cell renal carcinoma cells *in vitro*. FOXO6 differs from other FOXO proteins. Indeed, this protein is often highly expressed in tumor tissues and inhibits apoptosis, thus acting as an oncogene (32, 40). Additionally, using FOXO6-targeting small interfering RNA (siRNA) in HCC cells, Chen et al. (41) observed significantly increased apoptosis rates, increased P27 expression, decreased cyclin D1 expression, increased numbers of G_0_/G_1_-phase cells, and decreased numbers of S-phase cells (41).

**Table 1:** Downstream target genes of Forkhead box O proteins in tumor cells.

Target gene	Biological function
FasL	Apoptosis induced by cell death receptor
P27	Inhibition of proliferation/induction of apoptosis
BIM	Induction of the endogenous apoptosis pathway
BCL-6	Transcription repression
Cyclin B	Promotion of cell cycle completion
PIK	Promotion of cell cycle completion
GADD45	DNA repair
MnSOD	Protection of cells from peroxide damage
Catalase	Protection of cells from peroxide damage
TRAIL	Induction of apoptosis

FasL, Fas ligand; PIK, phosphatidylinositol kinase; GADD45, growth arrest and DNA-damage-inducible α; MnSOD, manganese superoxide dismutase; TRAIL, tumor necrosis factor-related apoptosis-inducing ligand.

### Roles of FOXO Proteins in Cancer Invasion and Metastasis

Compared with benign tumors, malignant tumors exhibit the unique characteristics of invasion and metastasis, which are important factors affecting treatment and prognosis. Tumor cells invade the extracellular matrix through cell–cell interaction and adhesion to the vascular endothelium; subsequently, invading cancer cells move to distant locations through osmotic movement and disseminate by forming new blood vessels (42). Cytokines are involved in tumor endothelial cell interactions, which occur between various surface adhesion molecules, including integrin, intracellular adhesion molecule-1, vascular cell adhesion molecule-1, or selectin (43). Zhang et al. (44) suggested that FOXO1 could limit the migration of prostate cancer cells by antagonizing Runt-related transcription factor 2. Kikuno et al. (45) demonstrated that the invasion of prostate cancer cells was associated with overexpression of astrocyte-elevated gene 1 (AEG1). Conversely, AEG1 gene knockout decreased cell invasiveness and increased FOXO3 expression (45). Therefore, the pro-invasion effect of AEG1 is associated with inhibition of FOXO3. Su et al. (46) reported that FOXO4 expression was decreased in gastric cancer tissues and cells compared with that in adjacent tissues and normal stomach cells. Moreover, FOXO4 upregulation inhibited the metastasis of gastric cancer cells and led to significant decreases in liver and lung metastasis *in vivo*, whereas FOXO4 downregulation with specific siRNA promoted the metastasis of gastric cancer cells (46).

Epithelial–mesenchymal transition (EMT) serves key roles in tumor invasion, metastasis, chemotherapy resistance, and tumor stem cell differentiation (47, 48). FOXO proteins can bind to downstream target DNA or protein to regulate the transcription or protein activity of targets such as Snail and β-catenin (47, 48). These molecules are important switches for EMT. The loss of E-cadherin is the first step in the EMT process (47, 48). FOXO1 can compete with E-cadherin for binding to miR-9, thus relieving the inhibition of E-cadherin by miR-9 (47). Moreover, Dong et al. (48) demonstrated that overexpression of FOXO1 could reverse the EMT process, inhibit cell motility and invasion *in vitro*, and suppress liver cancer lung metastasis *in vivo*. Bioinformatics results by Ni et al. (49) revealed that FOXO3a was associated with the metastasis of clear cell renal carcinoma, and its downregulation resulted in upregulated Snail and enhanced EMT (49). FOXO3a can directly bind to β-catenin, block its effects on downstream target molecules, inhibit EMT, and interfere with tumor malignancy (50). In a study of cholangiocarcinoma, FOXO4 was shown to mediate the EMT process, thereby affecting tumor progression and metastasis (51).

Alternatively, FOXO proteins can induce invasion and metastasis in some types of tumor. For example, cell division cycle 25A (CDC25A) directly regulates the transcription of matrix metalloproteinase 1 through FOXO1, thereby enhancing the invasive ability of breast cancer cells (52). In addition, CDC25A promotes breast cancer cell growth in mouse models (52). The simultaneous activation of β-catenin and FOXO3 can prevent the death and promote the metastasis of tumor cells (53). Arques et al. (54) found that β-catenin can resist the effects of PI3K/AKT inhibitors, thereby reactivating FOXO proteins, and high expression levels of FOXO3 and β-catenin in human colon cancer cells can lead to tumor metastasis and decreased patient survival. Furthermore, Sisci et al. (55) revealed the effects of FOXO3 on tumor cell invasion were closely associated with estrogen receptor (ER)α expression. In ERα^+^ cells, FOXO3 and 17β-estradiol acted synergistically to decrease invasive ability, whereas FOXO3 appeared to increase invasive ability in ERα^-^ cells. Wang et al. (40) reported that high FOXO6 expression in gastric cancer was associated with tumor invasion and poor prognosis. Additionally, overexpression of FOXO6 can inhibit tumor cell invasion and migration in colorectal cancer (31).

### Roles of FOXO Proteins in Tumor Resistance

Drug resistance remains one of the main obstacles in tumor treatment. Therefore, elucidating the molecular mechanisms of drug resistance and developing new molecularly targeted drugs are urgently required.

Studies on the roles of FOXO proteins in drug resistance have yielded positive results. For different types of tumors, changes in FOXO activity may reverse drug resistance. Additionally, PI3K inhibitors are effective against MEK inhibitor-resistant tumors, which may be associated with activation of FOXO3a transcription (56). Ausserlechner et al. (57) demonstrated that certain types of acute leukemia are resistant to prednisone due to inactivation of FOXO3a, which allows leukemic cells to escape the apoptotic effects of TRAIL and NOXA. Furthermore, the acetylation status of FOXO3a is a key factor in tumor resistance to cisplatin (58).

In addition to chemical inhibitors, siRNA has become a powerful tool for the study of tumor resistance. For example, Sun et al. (59) transfected colorectal cancer cells with siRNA to block the P85 and PI3K regulatory subunits, resulting in increased FOXO activity, cell cycle arrest, sensitivity to 5-fluorouracil, and reduced cell proliferation. Additionally, Shi et al. (60) observed that using siRNA to simultaneously silence the RAS and AKT oncogenes in pancreatic cancer cells *in vitro* had synergistic effects, resulting in increased apoptosis with decreased cell proliferation and clone formation. Furthermore, additional experiments with nude mice revealed that this treatment inhibited tumor growth (60).

FOXO proteins also serve roles in promoting tumor resistance. For example, FOXO proteins activated by PI3K/AKT inhibitors induce the activation of cell compensatory and adaptive mechanisms, leading to decreased drug sensitivity to trastuzumab and cell apoptosis (61). Trastuzumab can decrease the proliferation of breast cancer cells by inducing miR-542-3p expression (61). Moreover, PI3K inhibitors can downregulate miR-542-3p expression by activating FOXO1, thereby decreasing the efficacy of trastuzumab (61). In human epidermal growth factor receptor (HER)-2^+^ tumor cells with an overactivated PI3K/AKT signaling pathway, the activity of FOXO3 was promoted by AKT inhibitors, resulting in increased expression of tyrosine kinase receptors, such as HER3, IGF-1, and insulin receptors, as well as enhanced cell aggressiveness (62). The induction of mTOR by FOXO3 stimulates activation of AKT in renal cell carcinoma, while inhibition of FOXO3 activity enhances the sensitivity of renal cell carcinoma cells to PI3K and AKT inhibitors (63).

FOXO proteins can induce drug resistance through other mechanisms. The promoter region of multidrug resistance-associated protein 2 (MRP2) contains four FOXO-binding loci, and overexpression of MRP2 in tamoxifen-resistant breast cancer cells is closely associated with FOXO1 (64). In doxorubicin (DOX)-resistant breast cancer cells, FOXO1 induces therapeutic resistance by activating MRP1, whereas in K562 DOX-resistant leukemia cells, FOXO3 has been confirmed to be an important regulator of MRP1 (65, 66). Some antitumor drugs exert cytotoxic effects by increasing oxidative stress levels in tumor cells, and FOXO proteins can function to maintain cell stability by inducing the expression of antioxidant enzymes. For example, FOXO1 is overexpressed in paclitaxel-resistant ovarian cancer cells, which can protect these ovarian cancer cells from oxidative stress-induced apoptosis through regulation of superoxide dismutase 2 (SOD2) (67). In addition, activation of FOXO1 in esophageal squamous cell carcinoma upregulates cancer-associated fibroblasts and promotes the secretion of TGF-β1, thereby inducing cisplatin and paclitaxel resistance in esophageal squamous cell carcinoma cells (68).

### Association Between FOXO Expression and Cancer Prognosis

Several studies have revealed that FOXO proteins are independent factors affecting the prognosis of patients with cancer. FOXO1 mutations in B-cell non-Hodgkin’s lymphoma have been associated with a decrease in overall survival after patients received rituximab, cyclophosphamide, DOX, and prednisone chemotherapy treatments (69). Notably, phosphorylation of FOXO1 blocks the proliferation and angiogenesis of gastric cancer cells, improves the overall survival rate of patients with gastric cancer, and decreases tumor metastasis rates (70, 71). Moreover, overexpression of FOXO3 is associated with reduced overall survival and progression-free survival in patients with acute myelogenous leukemia and with poor prognosis in patients with pancreatic ductal adenocarcinoma and glioblastoma (72–74). Studies on the role of FOXO3 in breast and colorectal cancer have revealed that high FOXO3 expression in the nucleus predicts poor prognosis (53, 75). Li et al. (76) used univariate and multivariate Cox proportional-hazards models to assess survival risk factors and further verified the aforementioned results in cohorts from The Cancer Genome Atlas, demonstrating that FOXO4 could independently predict the overall survival of patients with gastric cancer. Low expression levels of FOXO3 and FOXO4, as well as lymph node metastasis, are risk factors for poor prognosis in patients with bladder cancer (77). FOXO6 overexpression is associated with poor prognosis in patients with gastric cancer and is observed in patients with serosal infiltration and lymph node metastasis (40).

### Roles of FOXO Proteins in Oxidative Stress

FOXO proteins serve important roles in defense against cellular oxidative stress. Oxidative stress damage is caused by the excessive accumulation or incomplete degradation of reactive oxygen species (ROS) (78–80). FOXO proteins effectively remove ROS, thereby decreasing their carcinogenic effects (9). Kops et al. (78) demonstrated that the anti-oxidative effects of FOXO proteins are achieved by increasing the expression levels of manganese SOD (MnSOD) and catalase. The role of FOXO proteins in regulating oxidative stress was confirmed by gene deletion. For example, Tothova et al. (79) found that mouse cells with FOXO gene knockout exhibited increased ROS levels and decreased antioxidant enzyme levels; further, ROS accumulation led to myelodysplastic syndrome in FOXO3^−/−^ mice. Ambrogini et al. (80) observed that expression of antioxidants such as glutathione was decreased in osteoblasts lacking FOXO1 expression.

Notably, stress stimulation can effectively counteract the inhibitory effects of AKT on FOXO proteins *via* the PI3K/AKT signaling pathway. For example, stimulation with growth factors, such as insulin-like growth factor, inhibits FOXO activity through phosphorylation *via* the PI3K/AKT signaling pathway. Under oxidative stress stimulation, JNK can phosphorylate 14-3-3 proteins, resulting in their dissociation from FOXO proteins. The phosphorylated 14-3-3 proteins are subsequently released into the extracellular matrix while the phosphorylated FOXO proteins enter the nucleus to induce transcription factor activity and mediate downstream gene transcription (12, 81, 82). When cells undergo oxidation *via* external stimuli, such as transportin-1, importin (IPO)7, or IPO8, FOXO proteins can form disulfide bonds with these molecules, which are then cotransported to the nucleus to mediate the transcription of genes that encode antioxidants, such as catalase, SOD2, glutathione peroxidase, and glutathione S-transferase-μ1, to maintain cell homeostasis (83–85).

### Signaling Pathways of FOXO Protein Regulation in Tumors

**Figure 2:**
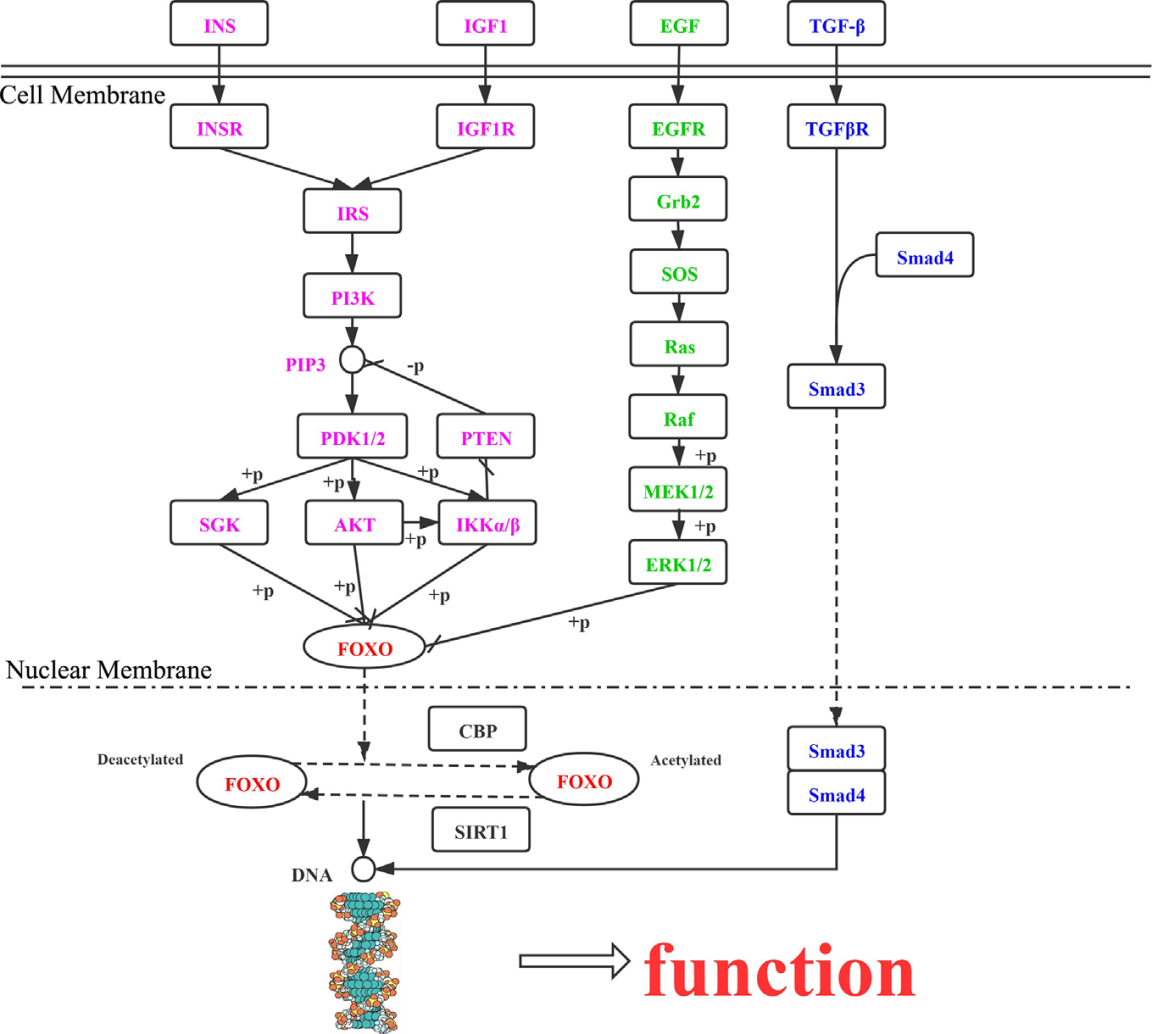
Roles of FOXO proteins in tumor cells, mediated *via* the PI3K/AKT, MAPK, IKK, and TGF-β signaling pathways. INS, insulin; INSR, insulin receptor; IRS, insulin receptor substrate; PIK3, phosphatidylinositol-4,5-bisphosphate 3-kinase; PDK, 3-phosphoinositide dependent protein kinase-1; AKT, RAC serine/threonine-protein kinase; SGK, serum/glucocorticoid-regulated kinase 1; IKK, inhibitor of nuclear factor κ-β kinase subunit α; PTEN, phosphatidylinositol-3,4,5-trisphosphate 3-phosphatase and dual-specificity protein phosphatase PTEN; IGF1R, insulin-like growth factor 1 receptor; IGF1, insulin-like growth factor 1; TGFβ1, transforming growth factor β-1; TGFβR1, TGF-β receptor type-1; SMAD4, mothers against decapentaplegic homolog 4; SMAD3, mothers against decapentaplegic homolog 3; EGF, epidermal growth factor; EGFR, epidermal growth factor receptor; GRB2, growth factor receptor-bound protein 2; SOS, son of sevenless; RAS, GTPase HRas; BRAF, B-Raf proto-oncogene serine/threonine-protein kinase; MEK1, mitogen-activated protein kinase kinase 1; ERK, mitogen-activated protein kinase 1/3; CBP, E1A/CREB-binding protein; SIRT1, NAD+-dependent protein deacetylase sirtuin 1.

In the cell cytoplasm and nucleus, activation and metastasis of FOXO proteins mainly rely on the PI3K/AKT, MAPK, IKK, and TGF-β signaling pathways. The effects of the different pathways on FOXO protein regulation are presented here (**Figure 2**).

### PI3K/AKT Pathway

FOXO proteins are downstream targets of the PI3K/AKT signaling pathway, whose function is suppressed by AKT through phosphorylation. When the PI3K/AKT pathway is abnormally activated, transcriptional deactivation of FOXO proteins promotes the proliferation of tumor cells (36). In response insulin and growth factor signaling, AKT/SGK can phosphorylate FOXO1 and FOXO3, which bind with 14-3-3 proteins in the nucleus, dissociate, and then translocate to the cytoplasm for transcriptional inactivation (14). Scheijen et al. (86) found that FLT3-ITD expression in acute myeloid leukemia activated the PI3K/AKT pathway, preventing FOXO3-induced apoptosis, upregulation of P27Kip1, and expression of BIM.

### MAPK Pathway

The MAPK signaling pathway includes ERK, JNK, and P38. Yang et al. (87) reported that FOXO3 downregulation resulted from ERK phosphorylating sites S294, S344, and S425, and FOXO3 degradation *via* the ubiquitin-proteasome-dependent pathway was counteracted by MDM2, thus causing tumor cell apoptosis. Sunayama et al. (88) determined that AKT and ERK were important in regulating FOXO3A levels in keratinoma, leading to tumors. Different from AKT and ERK, JNK and MST have functions in promoting the nuclear positioning of FOXO proteins and increasing their transcriptional activity. The activation of JNK and MST causes 14-3-3 protein phosphorylation, further enabling FOXO3a to dissociate from 14-3-3 proteins in the cytoplasm under oxidative stress (89, 90).

### IKK Pathway

The IKK kinase complex is important in the NF-κB pathway, as it enables hydrolysis of FOXO3a to promote tumor production by ubiquitination, revealing an interaction between the PI3K/AKT and NF-κB pathways (91). In addition, IKKϵ, another member of the IKK family, can phosphorylate FOXO3a to inhibit its activity (92).

### TGF-β Pathway

FOXO proteins are also involved in other cellular pathways. Zhao et al. (93) demonstrated that stimulation of hepatocytes with TGF-β resulted in FOXO proteins interacting with BIM and enhanced transcription of the target gene to promote apoptosis.

In summary, FOXO proteins are modulated under different conditions to determine cell cycle arrest, apoptosis, or stress resistance. At the same time, FOXO proteins are involved in a variety of signaling pathways related to tumor cell survival, and interactions occur between the pathways.

FOXO proteins are widely expressed in human body tissues, and their activities are adjusted by a series of post-translational modifications. FOXO proteins are mutually regulated with several carcinogenic signaling pathways, especially Insulin/PI3K/AKT signal pathway. Others include MAPK, IKK, TGF-β pathway and so on also are involved in the modifications. On one hand, FOXO proteins are widely studied as tumor suppressors by regulating cancer cells proliferation, apoptosis, invasion, metastasis, EMT, oxidative stress, etc. On the other hand, some subtypes of FOXO proteins have been found to promote tumor growth and maintenance of tumor survival, with the in-depth analysis in the functions of some tumors such as leukemia, breast cancer, colon cancer and HCC. In the clinical application, FOXO proteins could be the direct or indirect target in tumor treatment. The mutation or the activity by PI3K inhibitor of FOXO protein, activated FOXO proteins affect PI3K/AKT signal pathway through negative feedback regulation, to induce recurrence, metastasis and drug resistance of tumors, and relate closely with the occurrence, development, progress and prognosis. With the awareness of the double roles in the anti-tumor and promoting tumor of FOXO proteins, new ideas in tumor treatment would be provided for clinical therapeutics.

## Foxo Proteins In Hcc

**Figure 3:**
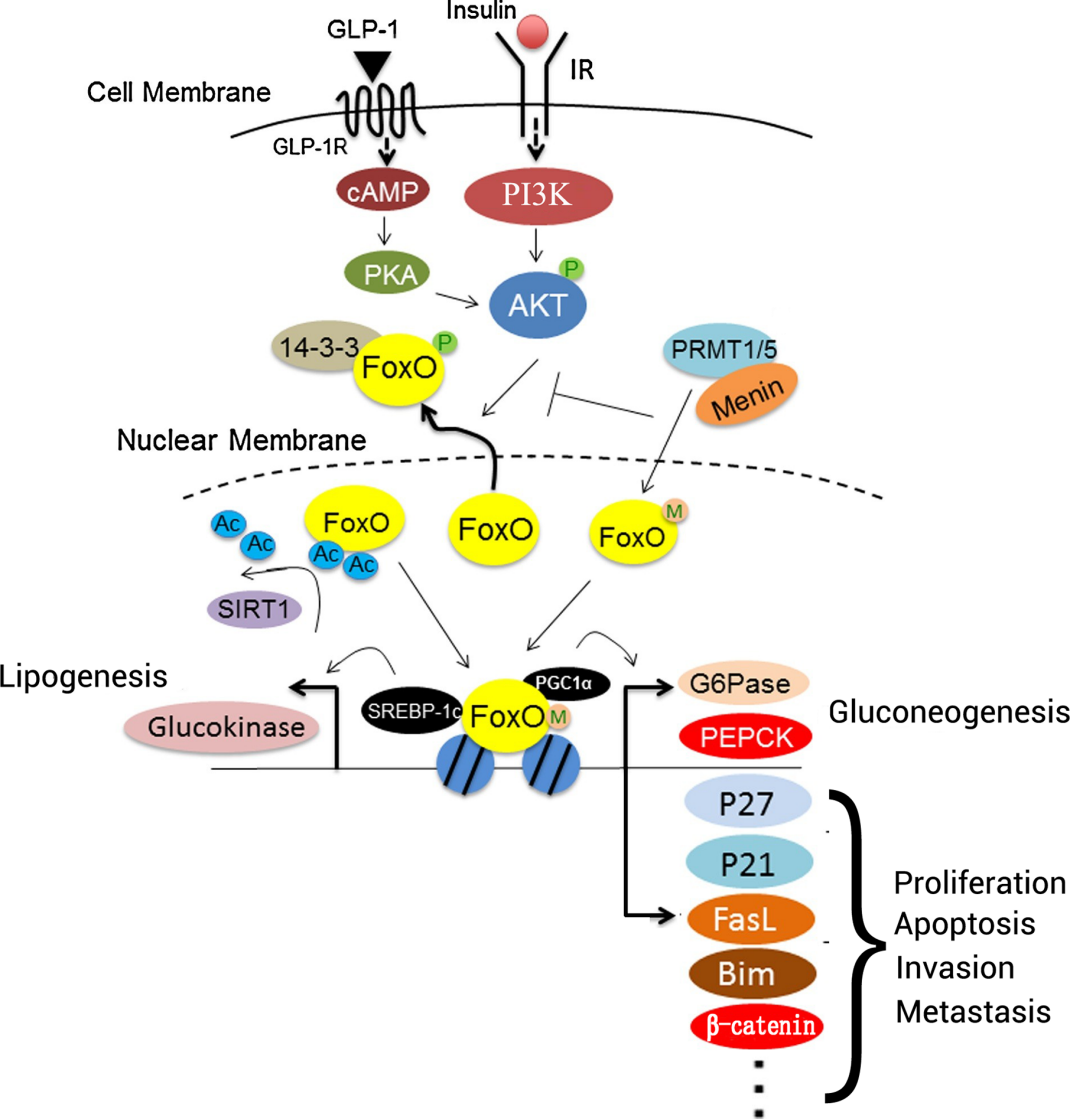
Roles of FOXO proteins in liver cells. Insulin or GLP-1 treatment can activate both PI3K/AKT and PKA. FOXO proteins interact with PGC1α to regulate the expression of gluconeogenesis-associated genes, including G6Pase and PEPCK, to promote gluconeogenesis. In addition, FOXO proteins interact with P27, P21, FasL, BIM, and β-catenin to regulate cell proliferation, apoptosis, invasion, and metastasis. SIRT1 deacetylates FOXO proteins after binding, which promotes their transcriptional activity. Menin may recruit PRMT1 or PRMT5 to methylate FOXO proteins, and it sterically hinders the phosphorylation of FOXO proteins by protein kinases. Additionally, FOXO proteins interact with SREBP-1c to activate the transcription of lipogenesis-associated genes involved in lipid metabolism, such as glucokinase. IR, insulin receptor; FasL, Fas ligand; FOXO, forkhead box O protein; GLP-1, glucagon-like peptide-1; GLP-1R, glucagon-like peptide-1 receptor; PEPCK, phosphoenolpyruvate carboxykinase; PGC1α, PRMT1, protein arginine methyltransferase 1; SIRT1, sirtuin 1; SREBP-1c, sterol regulatory element-binding protein 1; G6Pase, G6P-phosphatase; Ac, acetylation; PGC1α, peroxisome proliferator-activated receptor *γ* coactivator 1α; M, menin; P, phosphorylation.

Numerous studies have evaluated the roles of FOXO proteins, including FOXO1, FOXO3, FOXO4 and FOXO6, in HCC tumorigenesis, development, and prognosis (**Table 2** and **Figure 3**). Research concerning the PI3K/AKT pathway in HCC has been most abundant, but the MAPK, IKK, TGF-β pathways warrant further study with HCC-related interaction networks and enrichment analysis to elucidate the contributions of FOXO proteins in HCC.

**Table 2:** Effects of FOXO proteins on HCC tissues, cell function, treatment, and prognosis.

Process	FOXO1	FOXO3	FOXO4	FOXO6
Expression in HCC	Unknown	Increased	Unknown	Increased
Proliferation	Decreased	Increased	Unknown	Increased
Apoptosis	Increased	Decreased	Unknown	Decreased
Invasion and metastasis	Decreased	Increased	Unknown	Decreased
Tumor resistance	Decreased	Increased	Unknown	Increased
Prognosis	Unknown	Decreased	Unknown	Decreased

FOXO, forkhead box O protein; HCC, hepatocellular carcinoma.

During tumorigenesis and development, diverse signaling pathways interfere with FOXO proteins to change their expression, as well as their modification by phosphorylation and acetylation. Sirtuin 6 (SIRT6) combines and interacts with FOXO3 to regulate FOXO3 deacetylation, and it increases FOXO3 ubiquitination and phosphorylation at site S253, which is critical for FOXO3 degradation. Acetylation affects kinase binding to FOXO3 and leads to the apoptotic effects of FOXO3 that are independent of SIRT6 downregulation (94). TGF-β induces FOXO3 activation through specific de-phosphorylation at Thr32, which is regulated by casein kinase I-ϵ (CKI-ϵ) (93). TGF-β-activated FOXO3 then interacts with Smad2/3 to mediate BIM upregulation and apoptosis. After exposure to inflammatory lipopolysaccharide (LPS), the PI3K/AKT pathway is activated, FOXO6 is phosphorylated at Ser184, and downstream gene expression is inhibited (95). FOXO protein phosphorylation is involved in the diversification of upstream proteins in HCC cells; for example, TCF19 causes FOXO1 phosphorylation to enhance cell proliferation and tumorigenesis (96). However, the phosphorylation site of FOXO1 was not mentioned in these studies (**Figure 1**).

### FOXO1 in HCC

Production of excessive ROS can cause oxidative damage to cells. FOXO1 can shuttle between the nucleus and cytoplasm to resist oxidative stress (9), which mediates tumorigenesis. Jiang et al. (25) treated HCC cell lines with different concentrations of trifluoperazine, thereby blocking the cytoplasmic translocation of FOXO1 and increasing nuclear FOXO1 expression. These changes resulted in enhanced Bax/BCL-2 ratios and decreased expression levels of vascular endothelial growth factor, BCL-2, and proliferating cell nuclear antigen (PCNA), thereby leading to increased apoptosis and antitumor effects. Additionally, Li et al. (86) overexpressed aquaporin 9 in SMMC7721 HCC cells, resulting in increased FOXO1 expression, followed by downregulation of PCNA and upregulation of caspase-3 expression. Additionally, the cell cycle was arrested in the G_1_ phase to increase apoptosis and inhibit cell proliferation (97). Lee et al. (98) revealed that FOXO1 participated in growth arrest at the G_2_/M phase and promoted cell proliferation following RNA interference of Aurora kinase A. Moreover, Zeng et al. (99) demonstrated that EPS8-like 3 was highly expressed in HCC tissues and cells and promoted HCC cell proliferation by hyperactivating the AKT signaling pathway, and subsequently, inhibiting FOXO1 transcriptional activity. Kan et al. (100) suggested that zinc finger and BTB domain-containing 20 could be used as a prognostic marker for HCC and promoted the viability, proliferation, tumorigenicity, and cell cycle progression of HCC cells by transcriptionally inhibiting FOXO1. FOXO1 serves important roles in multiple signal transduction pathways, most notably the insulin/PI3K/AKT signaling pathway, which has negative regulatory effects on FOXO1. The activation of this pathway can increase cell survival, promote cell proliferation, and induce cancer development (12, 13). Jiang et al. (101) observed that the antithrombotic drug polydatin blocked the AKT/STAT3/FOXO1 signaling pathway in HCC cells, which increased the levels of phosphorylated AKT, phosphorylated Janus kinase 1, and STAT3, thereby blocking FOXO1. Thus, low FOXO1 expression promotes the apoptosis of HCC cells *via* G_2_/M arrest and inhibits the migration and invasion of HCC cells associated with EMT. Chiu et al. (102) suggested that the HBV X protein (HBX)-K130M/V131I double-mutant variant could promote HCC progression by activating the AKT/FOXO1 signaling pathway and inducing strong inflammation in the liver *via* arachidonic acid metabolism. Additionally, Dong et al. (48) demonstrated that overexpression of FOXO1 could inhibit cell migration and invasion *in vitro* by reversing the EMT process, as well as could inhibit liver cancer lung metastasis *in vivo*. Furthermore, Chi et al. (103) suggested that FOXO1 regulated BIM expression and indirectly downregulated thyroid hormone and its receptor, leading to chemotherapy resistance and DOX-dependent metastasis in hepatoma cells.

miRNA molecules are non-coding RNA transcripts 18–23 nucleotides long that regulate post-transcriptional gene expression by interfering with the translation of one or more target genes (104). Notably, miRNA can affect biological functions in tumors by positively or negatively affecting the target gene FOXO1. For example, Lin et al. (105) confirmed that upregulation of miR-5188 expression in patients with HCC modulated FOXO1 function, which interacted with β-catenin in the cytoplasm to decrease the nuclear transport of β-catenin and promote activation of Wnt signaling, tumor stemness, EMT, and c-Jun levels. Additionally, miR-196a (106, 107), miR-3174 (108), miR-1269 (109), miR-135b, and miR-194 (110) also target FOXO1, inducing varying effects. For example, FOXO1 is a direct target of miR-1269, and suppression of FOXO1 by miR-1269 is associated with dysregulation of p21, cyclin D1, and expression of phosphorylated RB and Ki67 (109).

### FOXO3 in HCC

The main mechanism regulating the activity of FOXO3 and its target genes involves controlling the nuclear-cytoplasmic shuttling of FOXO3. This process can be achieved *via* the phosphorylation of a series of kinases, such as PKB, ERK, serum- and glucocorticoid-inducible kinase 1 (SGK1), and IKKβ to promote the nuclear export of FOX03a (111).

In a study by Ahn et al. (21), 121 (64.71%) HCC cases exhibited high FOXO3a expression levels, which was associated with aggressive phenotypes of HCC and poor survival rates. Notably, the inhibition of FOXO3a in HepG2 cells inhibits cell proliferation and migration (21). Song et al. (20) demonstrated that FOXO3 expression was significantly increased in HCC samples compared with that in non-cancerous liver tissue samples, and FOXO3 expression was significantly associated with tumor metastasis, α-fetoprotein (AFP) level, and overall survival rate. Therefore, high FOXO3 expression may predict poor prognosis in patients with liver cancer. Lu et al. (112) reported that FOXO3 was significantly activated in pathological specimens from patients with HCC. In addition, FOXO3 activated positive feedback pathways, such as the PI3K/AKT and mTOR complex 2 signaling pathways, in the hepatocytes from a transgenic mouse model with high FOXO3 expression, thereby promoting oxidative stress and DNA damage to regulate ROS (20). Alternatively, FOXO3 can activate the pentose phosphate pathway to form ROS-eliminating systems. Thus, personalized analyses of the activation state of FOXO3 in patients with HCC are needed. Tao et al. (113) demonstrated that inhibition of FOXO3 through phosphorylation of β-catenin induced by SGK1 decreased the pro-apoptotic function of FOXO3, resulting in increased hepatocyte survival. Li et al. (33) demonstrated that circular RNA FBXO11 was predominantly localized in the cytoplasm and bound to miR-605, which targeted FOXO3, thereby promoting HCC proliferation, cell cycle progression, and oxaliplatin resistance. Qian et al. (114) suggested that downregulation of inhibitor of growth 4, which targeted the NF-κB/miR-155/FOXO3a signaling pathway, was closely associated with cancer staging, tumor size, and vascular invasion in HCC. Using HepG2 cells, Kim et al. (115) demonstrated that *Houttuynia cordata* induced apoptosis by targeting and increasing FOXO3 expression. Additionally, Kim et al. (115) found that ergosterol peroxide could activate FOXO3-mediated cell death signaling by inhibiting AKT and c-Myc. Notas et al. (116) observed that the binding of a proliferation-inducing ligand to B-cell maturation antigen activated the JNK2/FOXO3/GADD45 signaling pathway and induced G_2_/M arrest. Furthermore, Hu et al. (94) reported that high SIRT6 expression and low FOXO3 expression after DOX treatment led to TACE resistance. In subsequent experiments, overexpression of SIRT6 did not prevent DOX-induced death in the context of FOXO3-knockdown (94). As an effective chemotherapeutic agent for advanced HCC, DOX can downregulate SIRT6 expression, transport FOXO3 to the nucleus, and upregulate FOXO3 expression (94). During this process, SIRT6 interacts with FOXO3, thereby promoting FOXO3 ubiquitination and decreasing its stability (94). Moreover, P27 and BIM expression is increased to induce cell apoptosis (94). Gao et al. (117) demonstrated that FOXO3, as a polycomb group protein-related target gene involved in DNA damage, was associated with increased drug sensitivity in HCC cells. Moreover, Lin et al. (118) suggested that FOXO3 was an important target of methyltransferase-like 3-mediated m6A modification in the resistance of HCC to sorafenib therapy and could activate autophagy-associated signaling pathways.

### FOXO4 in HCC

FOXO4 activity can be regulated by both classical phosphorylation and acetylation. Previous studies have revealed that mono-ubiquitination (119) and O-linked N-acetylglucosamine glycosylation (120) are important post-translational modifications of FOXO4, serving key roles in its biological functions. FOXO4 is a downstream target of the PI3K/AKT signaling pathway, and AKT changes the function of FOXO4 *via* phosphorylation (36). In tumor cells, the PI3K/AKT signaling pathway is abnormally active, resulting in changes in FOXO4 expression. Liou et al. (121) reported that curcumin could induce the translocation of FOXO4 from the cytoplasm to the nucleus through the AKT/PTEN/FOXO4 signaling pathway and induce apoptosis in P53-invalid liver cancer cells. Furthermore, Gong et al. (122) found that non-structural maintenance of chromosome condensin I complex subunit G (NCAPG) acted as an oncogene in liver cancer, as it promoted cell proliferation and inhibited apoptosis by activating the PI3K/AKT/FOXO4 signaling pathway. Additionally, Li et al. (123) reported that benzo[a]pyrene could induce pyroptotic and autophagic death by blocking the PI3K/AKT signaling pathway in liver HL-7702 cells and inhibiting the phosphorylation of FOXO4.

Cui et al. (124) revealed that ROS from NOX1/NADPH oxidase can oxidize and inactivate PTEN, thereby positively regulating the AKT/FOXO4/P27(kip1) signaling pathway, promoting hematopoietic stem cell proliferation following liver injury induced by bile duct ligation and accelerating the development of liver fibrosis. Moreover, You et al. (125) reported that YH0618 promoted viability and inhibited DOX-induced apoptosis of normal L02 hepatocytes through a mitochondrial-dependent mechanism mediated by FOXO4, and it reduced the toxicity caused by DOX. Yuan et al. (126) demonstrated that isoorientin, a flavonoid compound extracted from several plant species, inhibited AKT phosphorylation and increased FOXO4 expression through mitochondrial dysfunction and the PI3K/AKT signaling pathway in HepG2 cancer cells, thereby inducing cell death in a concentration-dependent manner. This process was not toxic to normal liver cells.

HBV is the main cause of chronic hepatitis and HCC (2). Fu et al. (127) demonstrated that HBV could promote miR-328-3p expression through the STAT3 signaling pathway and downregulate its target, FOXO4, resulting in cell damage to THLE-2 hepatocytes infected with HBV. Additionally, Srisuttee et al. (128) indicated that upregulation of FOXO4 expression mediated by HBX enhanced resistance to cell death induced by oxidative stress. HBX is a regulatory protein of HBV and produces ROS in human liver cell lines (128). HBX induces FOXO4 upregulation in Chang cells that stably express HBX (Change-HBX cells) and in primary liver tissues from HBX transgenic mice (128). Additionally, HBX increases ROS levels; however, treatment with N-acetylcysteine to decrease ROS content also blocks FOXO4 expression (128). FOXO4 siRNA-mediated silencing increases Chang-HBX cell sensitivity to apoptosis under oxidative stress (128). Furthermore, Wang et al. (129) reported that upregulation of coiled-coil domain containing 50 is regulated by the HBX/splicing factor, arginine/serine-rich 3/14-3-3β complex, and that it enhances the carcinogenicity of HCC through the Ras/FOXO4 signaling pathway. Destruction of the intestinal barrier is known to cause endotoxins and other bacterial products in the intestinal lumen to enter circulation, leading to hepatitis and liver injury (129). Chang et al. (130) revealed that FOXO4 is involved in epithelial barrier dysfunction. In their study, TNFα was found to upregulate phosphorylation of FOXO4, leading to nuclear localization and inactivation of the protein; FOXO4 then lost the ability to inhibit NF-κB activity, downregulated the expression levels of tight junction proteins, and increased epithelial permeability.

### FOXO6 in HCC

FOXO6 is the most recently identified member of the FOXO family and was initially only detected in mammalian brain tissues (9). However, Kim et al. (131) demonstrated that FOXO6 is also expressed in other tissues, including the liver, intestine, lung, kidney, muscle, and fat. Previous studies on FOXO6 have primarily focused on the regulation of glucose homeostasis in the liver. However, some studies have demonstrated that FOXO6 serves important roles in the occurrence and development of gastric cancer (40), lung cancer (132), and HCC (15, 41, 133). Chen et al. (41) revealed that FOXO6 is highly expressed in HCC tissue. Additionally, siRNA knockdown of FOXO6 increased P27 expression, decreased cyclin D1 expression, enhanced apoptosis rates, increased the number of cells in the G_0_/G_1_ phase, and decreased the number of cells in the S phase. Further analyses demonstrated associations among tumor size, tumor-node-metastasis (TNM) stage, AFP level, hepatitis B surface antigen status, degree of differentiation, and FOXO6 expression. Zuo et al. (133) found that 5-hydroxytryptamine receptor 1D (5-HT1D) and P85α (PIK3R1) could enhance FOXO6 expression through the PI3K/AKT signaling pathway. Furthermore, FOXO6 was directly transcriptionally activated by 5-HT1D in an AKT-independent manner. During this process, 5-HT1D stabilized PIK3R1 by inhibiting ubiquitin-mediated degradation and promoted the proliferation, EMT, and metastasis of HCC *in vitro* and *in vivo*.

Yu et al. (15) demonstrated that FOXO6 was expressed at high levels in HCC cells and that FOXO6 knockdown blocked the proliferation and invasion of HCC cells and induced their apoptosis. Notably, FOXO6 inhibited glycolysis, reversed chemotherapy resistance in Hep3B cells treated with paclitaxel, and inhibited the PI3K/AKT signaling pathway. After the PI3K/AKT signaling pathway is activated by 740Y-P (a PI3K activator) or inhibited by LY294002 (a PI3K inhibitor), FOXO6 knockdown affects resistance to paclitaxel chemotherapy, cytotoxicity, and glycolysis in HCC cells. Zhong et al. (134) used oxygen and glucose deprivation of L02 cells to generate a model of liver injury caused by oxidative stress in ischemia, revealing that FOXO6 regulates nuclear factor erythroid 2-related factor 2 through c-Myc and participates in isoflurane pre-conditioning as a stimulus factor to protect against liver injury. Kim et al. (95) demonstrated that FOXO6 promoted liver oxidative stress resistance and inhibited pro-inflammatory mediators, while serving protective roles in the maintenance of cell homeostasis under pro-inflammatory conditions induced by LPS. FOXO6 phosphorylation *via* the AKT and PAK1 signaling pathways facilitates the nuclear translocation of NF-κB induced by LPS (95). Additionally, Kim et al. (135) revealed that FOXO6-mediated IL-1β participated in hepatic inflammation and insulin resistance through the tissue factor/protease activated receptor 2 (PAR2) signaling pathway in the liver of FOXO6-knockout mice. FOXO6 expression is elevated in the livers of insulin-resistant aging rats and obese mice. FOXO6 directly binds to and increases the expression levels of IL-1β, thereby increasing the level of PAR2 and decreasing hepatic insulin signaling, while treatment with PAR2-siRNA eliminates these effects (135).

## Prospects

### FOXO Proteins as HCC Tumor Markers

Multiple experiments with HCC tissues and cells have shown that the occurrence and development of HCC is related to the expression of FOXO proteins, which is also positively correlated with the patient’s clinical stage, disease progression, prognosis and some clinical, pathological, or prognostic indicators. On the one hand, the widely studied FOXO1 regulates cellular function as a tumor inhibitor; on the other hand, FOXO3 and FOXO6 play important roles in promoting tumor growth and invasion and maintaining tumor survival.

FOXO1 inhibits the invasion and metastasis of HCC cells by reversing ZEB2-induced EMT, and low expression of FOXO1 exacerbates cell proliferation and apoptosis, thus driving pathogenesis (48, 136). In addition, FOXO1 expression differs in precancerous lesions from two conditions that lead to HCC, non-alcoholic steatohepatitis (NASH) and alcoholic steatohepatitis (ASH), which also reflects different tumorigenic rates for ASH and NASH (137). Overexpression of FOXO3a inhibits the proliferation, tumorigenic potential, and invasiveness of HCC cells, while silencing of FOXO3a markedly attenuates protection against tumorigenesis (21, 138). FOXO3 expression is also significantly increased in HCC samples compared with non-cancerous liver tissue samples. Further, FOXO3 expression is significantly associated with metastasis, TNM stage, Edmondson grade, AFP level, overall survival, and small vessel invasion. Moreover, high FOXO3 expression predicts poor prognosis in patients with HCC, indicating its potential as a therapeutic target for HCC (20, 21). FOXO6 is highly expressed in HCC cells compared with normal human hepatocytes, and FOXO6 knockdown inhibits the proliferation and invasion and induced apoptosis of HCC cells (15, 41). The expression of FOXO6 in HCC tissues is significantly higher than that in normal and adjacent HCC tissues and is related to tumor size, TNM stage, AFP level, the presence or absence of HbsAg, and differentiation degree (41).

### FOXO Proteins as Drug Targets

FOXO proteins play key roles in the occurrence of tumors and signal feedback of cellular factors; thus, inhibiting the activity of FOXO proteins appears to be the reasonable strategy for cancer treatment. PI3K inhibitors targeting the PI3K/AKT/FOXO pathway help prevent drug resistance, which may interfere with FOXO-induced apoptosis and block the cell cycle. However, the proliferation signals triggered by the PI3K pathway not only rely on FOXO proteins, but also directly induce strong anti-apoptosis and proliferative effects (139).

AS1842856 is a targeted inhibitor that can prevent FOXO1 translocation to the nucleus, which blocks Lapateni resistance of MYC-induced breast cancer (140). In addition to the small molecule inhibitors above, researchers have also developed two polypeptides with FOXO inhibitory function. The FOXO1 analog peptide can be combined with IQGAP1 as endogenous FOXO1, inhibiting feedback activation of ERK upon transfection of FOXO1, and improving the sensitivity of prostate cancer cells to PI3K inhibitors and paclitaxel (141). Therefore, inhibiting the activity of FOXO1 does not only cancel the therapeutic effect of the PI3K inhibitor but also helps prevent the occurrence of drug resistance. Another analog peptide of FOXO4 prevents the binding of FOXO4 and P53, inducing apoptosis and removing aging cells, but its role in tumors remains unclear (142).

Several inhibitors may not only target the PI3K/AKT/FOXO pathway, but also directly target FOXO proteins, thereby affecting HCC carcinogenic function and drug resistance. Polydatin, extracted from *Polygonum cuspidatum*, is known for its anti-platelet aggregation and anti-inflammatory effects. Polydatin may promote HCC cell apoptosis as a PI3K inhibitor by blocking the AKT/STAT3/FOXO1 signaling pathway (101). LY294002, a PI3K inhibitor, eliminates the role of NCAPG in promoting HCC cell proliferation, while 740Y-P, a PI3K activator, resists the effects of FOXO6 knockdown on the cytotoxicity and glycolysis of paclitaxel in HCC cells (15, 122). Isoorientin (ISO), a flavonoid compound that can be extracted from species such as *Phyllostachys pubescens*, *Patrinia* sp., and *Drosophyllum lusitanicum*, induces apoptosis mediated by mitochondrial dysfunction through the PI3K/AKT/FOXO4 signaling pathway (126).

Patients with schizophrenia tend to have reduced incidence of some cancers due to their treatment with antipsychotic drugs that have antitumor effects, such as trifluoperazine (TFP). TFP increases the nuclear localization of FOXO1 to restrict angiogenesis and tumor growth (25). Thyroid hormone (TH), a potent hormone mediator of cellular differentiation and metabolism, acts as an anti-apoptosis factor upon challenge of TH receptor (TR)-expressing HCC cells with cancer therapy drugs mediated by FOXO1 (103). *Houttuynia cordata* Thunb is a medicinal plant that enhances HIF-1A/FOXO3 signaling, leading to MEF2A upregulation to inhibit cell growth and induce cell apoptosis (115). Ergosterol peroxide (5α, 8α-epidioxiergosta-6, 22-dien-3β-ol), purified from *Ganoderma lucidum*, stimulates FOXO3 activity to induce HCC cell death (143). PS341 (Bortezomib) is the first proteasome inhibitor drug which has been approved for clinical treatment of multiple myeloma. PS341 upregulates FOXO3 to inhibit transcriptional activation of CTNNB1, leading to inhibition of HCC (144). Treatment with curcumin, a yellow-colored polyphenol with antiproliferative and proapoptotic activities, induced translocation of FOXO4 protein from the cytosol into the nucleus in P53-Null HCC cells to induce apoptosis (121). The medicinal and edible formula YH0618 reduces DOX-induced toxicity, especially with respect to the amelioration of effects of alopecia, and attenuates DOX-induced apoptosis in normal liver cells through a FOXO4-mediated mitochondria-dependent mechanism (125). CKI inhibition by small molecule D4476 abrogates TGF-β-induced FOXO3/Smad activation, reverses BIM upregulation, and blocks subsequent apoptosis (93). Studies have found both direct and indirect relationships between HCC and FOXO proteins. By investigating this relationship, we expect to develop new drugs for clinical treatment to mitigate and even cure HCC.

## Conclusion

Sorafenib is currently the only targeted drug approved for the treatment of patients with HCC. Although fundamental research and clinical studies have been conducted on alternative first- and second-line treatments, the results have generally been unsatisfactory. Recent studies have shown that epigenetic processes, such as DNA methylation, as well as miRNA expression, and the tumor microenvironment are associated with the occurrence and development of HCC. Angiogenic pathways and cytokine cascades are still major focuses of research in HCC (145). Overall, FOXO proteins serve important roles in tumorigenicity, tumor cell survival, tumor metastasis, and tumor drug resistance among various tumor tissues and cells. Moreover, FOXO proteins participate in tumor escape from immune surveillance and regulate the level of cellular oxidative stress. The study of FOXO proteins in HCC is detailed and meticulous. Two subtypes, FOXO3 and FOXO6, have been thoroughly studied and have been shown to play important roles in the development, prognosis, and treatment of HCC. Conversely, FOXO1 expression in HCC and its association with prognosis remain unclear. Although the involvement of FOXO4 has been investigated in tumor development as a downstream target of the PTEN/PI3K/AKT signaling pathway, few reports have discussed FOXO4 expression in HCC and its association with survival, prognosis, and drug resistance in patients with cancer. Thus, further bioinformatics analyses and basic experiments are required to fully elucidate the roles of these proteins in different types of cancer, including HCC. FOXO1, FOXO3, FOXO4, and FOXO6 may have applications as predictors of HCC progression and may be useful in future targeted gene therapy.

## Author Contributions

Among the authors in the list, SY, SW and JK designed the study and revised the manuscript. LP, WD, TR, YD, YZ, SB and XZ performed the literature search. SY drafted the manuscript. All authors contributed to the article and approved the submitted version.

## Funding

The present study was supported by The National Natural Science Foundation of China (grant no. 81670580), The Shenyang Science and Technology Innovation Talent Support Program for Youth and Midlife (grant no. RC200121) and The 345 Talent Project Program of China Medical University Shengjing Hospital (grant no. 2019-40A).

## Conflict of Interest

The authors declare that the research was conducted in the absence of any commercial or financial relationships that could be construed as a potential conflict of interest.
